# Transcutaneous Auricular Neurostimulation (tAN): A Novel Adjuvant Treatment in Neonatal Opioid Withdrawal Syndrome

**DOI:** 10.3389/fnhum.2021.648556

**Published:** 2021-03-08

**Authors:** Dorothea D. Jenkins, Navid Khodaparast, Georgia H. O’Leary, Stephanie N. Washburn, Alejandro Covalin, Bashar W. Badran

**Affiliations:** ^1^Department of Pediatrics, Medical University of South Carolina, Charleston, SC, United States; ^2^Spark Biomedical, Inc., Dallas, TX, United States; ^3^Department of Psychiatry & Behavioral Sciences, Brain Stimulation Division, Medical University of South Carolina, Charleston, SC, United States

**Keywords:** neonatal opioid withdrawal syndrome (NOWS), non-invasive neuromodulation, opioids, morphine, transcutaneous auricular neurostimulation, tAN, bioelectronic medicine

## Abstract

Maternal opioid use during pregnancy is a growing national problem and can lead to newborns developing neonatal opioid withdrawal syndrome (NOWS) soon after birth. Recent data demonstrates that nearly every 15 min a baby is born in the United States suffering from NOWS. The primary treatment for NOWS is opioid replacement therapy, commonly oral morphine, which has neurotoxic effects on the developing brain. There is an urgent need for non-opioid treatments for NOWS. Transcutaneous auricular neurostimulation (tAN), a novel and non-invasive form of electrostimulation, may serve as a promising alternative to morphine. tAN is delivered *via* a multichannel earpiece electrode worn on and around the left ear, targeting two cranial nerves—the vagus and trigeminal nerves. Prior research suggests that auricular neurostimulation exerts an anxiolytic effect on the body by releasing endogenous opioids and reduces withdrawal symptoms in adults actively withdrawing from opioids. In this first-in-human prospective, open-label trial, we investigated tAN as an adjuvant to morphine therapy in eight infants >33 weeks gestational age suffering from NOWS and receiving oral morphine treatment. Infants received tAN for 30 min 1 h before receiving a morphine dose. tAN was delivered at 0.1 mA below perception intensity at two different nerve targets on the ear: Region 1, the auricular branch of the vagus nerve; and Region 2, the auriculotemporal nerve. tAN was delivered up to four times daily for a maximum of 12 days. The primary outcome measures were safety [heart rate monitoring, Neonatal Infant Pain Scale (NIPS), and skin irritation] and morphine length of treatment (LOT). tAN was well-tolerated and resulted in no unanticipated adverse events. Comparing to the national average of 23 days, the average oral morphine LOT was 13.3 days (median 9 days) and the average LOT after tAN initiation was 7 days (median 6 days). These preliminary data suggest that tAN is safe and may serve as a promising alternative adjuvant for treating NOWS and reducing the amount of time an infant receives oral morphine.

## Introduction

Neonatal opioid withdrawal syndrome (NOWS) is a condition in which infants experience withdrawal symptoms after *in utero* exposure to prescription or non-prescription opioids such as methadone or heroin (Witt et al., 2017). Due to abrupt termination of opioid delivery to the central nervous system at birth, newborns typically experience withdrawal symptoms within 48–72 h (Kocherlakota, 2014), including tachycardia, tremors, high-pitched crying, tachypnea, vomiting, weight loss, mottling, dysregulation of body temperature, and disrupted sleep (Kraft and van den Anker, 2012; Ko et al., 2017; Patrick et al., 2020).

Treatment of NOWS usually follows a multi-modal regime centered on controlled withdrawal and replacement drug therapy with oral morphine. Although there is no nationwide standard of care, oral morphine or methadone are considered as the first-line therapy. Other drugs, including clonidine and phenobarbital, are used as adjuncts when needed. This approach is not optimal, as it can impart additional stress on the newborn (Hudak and Tan, 2012). Additionally, these pharmacotherapies themselves produce harmful side effects. Administering morphine during early neurodevelopment, specifically during a critical perinatal period, may cause neuronal apoptosis, white matter injury, decreased myelin maturation, and oxidative stress, leading to long-term developmental consequences (Rao et al., 2007; Attarian et al., 2014; Steinhorn et al., 2015; Flannery et al., 2020).

The American Academy of Pediatrics recommends attempting the use of non-pharmacologic treatment, which includes placing the infant in a dark and quiet environment, swaddling, rocking, breastfeeding, and providing high-calorie nutrition in frequent small feedings, among other techniques (Hudak and Tan, 2012; Grossman et al., 2018). When utilized appropriately, such non-pharmacological interventions have resulted in a reduction in length of stay, length of treatment (LOT), and percentage of infants requiring pharmacotherapy (MacMillan et al., 2018). With NOWS babies already under stress from opioid withdrawal, a non-pharmacological treatment may greatly benefit these patients, lowering the need for additional medications and potentially reducing their hospital stay.

Considering the hyperactivation of the sympathetic nervous system in NOWS (Jansson et al., 2010), activation of counterregulatory parasympathetic nerves *via* the release of acetylcholine might be beneficial (Janssen et al., 2011; Hu et al., 2021). Recently, a non-invasive form of vagus nerve stimulation (VNS) known as transcutaneous auricular VNS (taVNS) targets the auricular branch of the vagus nerve (ABVN) and activates vagal afferent and efferent networks (Kraus et al., 2013; Garcia et al., 2017; Yakunina et al., 2017; Badran et al., 2018a; Badran et al., 2019; Kaniusas et al., 2019). In addition to auricular vagal nerve branches, the ear is innervated by other cranial nerve branches such as the auriculotemporal nerve (the branch of the trigeminal nerve located superficial to the temporal mandibular joint). Stimulation of the ABVN at lower frequencies releases CNS endorphins (Sator-Katzenschlager and Michalek-Sauberer, 2007), inhibits the release of pro-inflammatory cytokines that augment pain (Chen et al., 2015), and has been shown to have sustained antinociceptive effects in multiple studies including post-operative studies of opioid intake (Sator-Katzenschlager et al., 2004; Kovacic et al., 2017). Animal and human research suggest that endogenous opioids may supplant the need for exogenous opioids, thus leading to antinociception and mitigation of opioid withdrawal symptoms (Liu et al., 1999; Wu et al., 1999; Han, 2004; Bonnette, 2008; Meade et al., 2010; de Andrade et al., 2011; He et al., 2011; Van Bockstaele and Valentino, 2013; Toubia and Khalife, 2019). In previous studies, auricular neurostimulation decreased symptoms of acute opioid withdrawal in adults (Severson et al., 1977; Wen et al., 1978; Ellison et al., 1987; Miranda and Taca, 2018). In neonates, alternative medicine approaches to auricular neurostimulation have recently been studied as adjunctive therapies for NOWS. Acupuncture (Filippelli et al., 2012; Raith et al., 2015), acupressure stickers, and laser acupuncture have been applied resulting in a reduction in withdrawal symptoms in newborns with NOWS. These results suggest that activating auricular neural pathways may have promising clinical effects and serve as adjunctive therapy for NOWS.

As various methods of auricular neuromodulation demonstrate a reduction in withdrawal symptoms and activate parasympathetic pathways important in autonomic regulation, our scientific premise is that tAN, when delivered before administration of oral morphine, may release endogenous opioids and reduce withdrawal symptoms, resulting in reduced LOT with oral morphine. In this first-in-human trial, we aimed to explore the safety and feasibility of delivering tAN as adjunctive therapy to oral morphine replacement to reduce the signs and symptoms associated with NOWS in neonates.

## Materials and Methods

### Study Overview/Design

**Figure 1:**
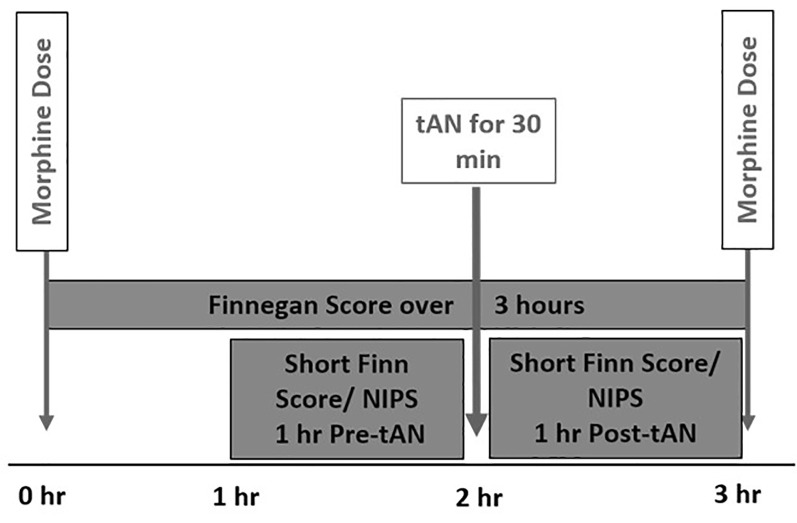
Incorporation of transcutaneous auricular neurostimulation (tAN) therapy with scheduled morphine therapy.

This first-in-human prospective, open-label trial was approved by the Medical University of South Carolina (MUSC) Institutional Review Board and was conducted at MUSC Shawn Jenkins Children’s Hospital in Charleston South Carolina. Parental consent was obtained by the study team before any study-related activities. Eight infants who were diagnosed with NOWS and receiving oral morphine replacement therapy were enrolled between May and December 2020. All infants received tAN in addition to morphine replacement therapy. tAN stimulation was delivered 1 h before each scheduled morphine dose during the peak withdrawal period (Figure 1). Primary outcome measures included safety (heart rate decrease, bradycardia, skin irritation, pain scores) and morphine LOT.

### Participants

Infants >33 weeks gestational age at enrollment with opioid dependence receiving oral morphine and on minimal respiratory support were included in this open-label clinical trial. Infants were excluded if they had any of the following: unstable respiratory or cardiovascular status or requiring significant respiratory support, repeated autonomic instability (apnea or bradycardia) which was not self-resolving, major unrepaired congenital anomalies impacting the respiratory or cardiovascular system, cardiomyopathy, and abnormal ear anatomy not allowing the device to fit.

### Opioid Replacement Therapy and Withdrawal Symptom Scoring

NOWS withdrawal symptoms include hyperirritability, hypertonicity, high-pitched crying, tachypnea, gastrointestinal dysfunction, and sleep disturbances. The Finnegan Neonatal Abstinence Scoring Tool (FNAST) is a validated assessment tool designed to measure 21 signs of withdrawal in infants (Finnegan et al., 1975; Devlin et al., 2020). The tool provides a means to rate the severity of withdrawal symptoms every 3 h after feeding using a standard format. In addition to the standard FNAST, a simplified FNAST scale with a high statistical correlation to the standard FNAST was used as part of the study protocol (Gomez Pomar et al., 2017; Devlin et al., 2020). This score was recorded by neonatal nurses 1 h before and after each tAN session to measure the immediate effects of tAN on withdrawal symptom severity.

The oral morphine administration protocol for NOWS at MUSC Shawn Jenkins Children’s Hospital initiates treatment with an oral morphine dose of 0.05 mg/kg/dose q 3 h, depending on clinical circumstances. If withdrawal symptoms remain well-controlled, defined as FNAST scores <8 for 72 h on a set dose of morphine, that dose is established as the “control dose,” and is the starting point for morphine weaning. In the standard protocol, morphine is weaned by 15% every 24–48 h if FNAST scores remain <8 for 24 h.

### Transcutaneous Auricular Neurostimulation (tAN) Adjuvant Protocol

After consent, the study team began administering tAN as an adjuvant therapy to morphine, four times per day, 1 h before morphine dose, for up to 12 days or the discontinuation of morphine (whichever came first). tAN administration began either, within the 72-h control dose period or after starting morphine weaning, for all infants enrolled. We followed an accelerated morphine weaning protocol for this study, weaning morphine every 12 h if FNAST scores remained <8 after tAN treatments were started. If after weaning or discontinuing morphine the infant had two consecutive FNAST scores >12 or three consecutive FNAST scores >8, an increase in dose or a rescue dose of morphine equal to the lowest dose before discontinuing morphine was given.

### Transcutaneous Auricular Neurostimulation (tAN) Overview

**Figure 2:**
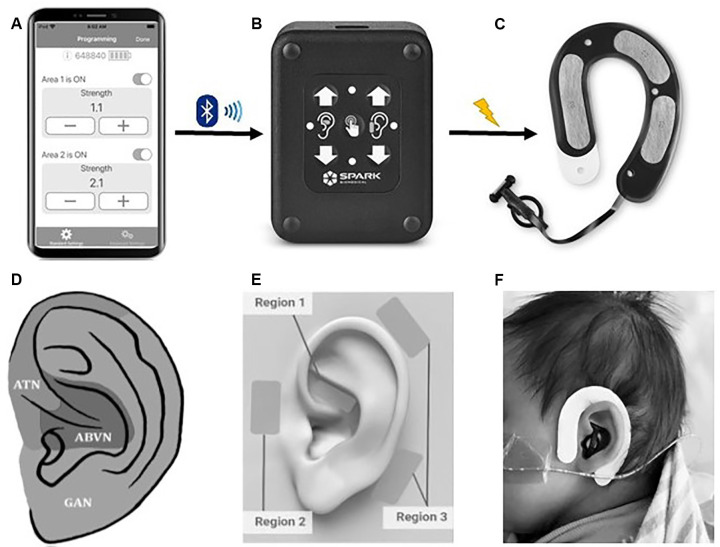
The Roo™ therapy system. **(A)** The clinician application connects an iOS device to the patient controller using bluetooth low energy. The application allows a clinician to start a communication session with a Patient Controller to adjust therapy parameters, view diagnostic logs, and event history. **(B)** The Patient Controller delivers electrical stimulation to the earpiece *via* a removable cable. Clinicians can modulate therapy intensity by pressing up/down buttons and check therapy status with LED lights. **(C)** The earpiece (skin-facing side) delivers electrical stimulation *via* four metallic electrodes covered in hydrogel and surrounded with adhesive hydrocolloid (black region). **(D)** Hypothesized auricular dermatomes. ATN, auriculotemporal nerve; ABVN, auricular branch of the vagus nerve; GAN, greater auricular nerve. Modified from Butt et al. (2020). **(E)** The Roo system provides stimulation in a bipolar configuration forming stimulation channels/circuits between pairs of electrodes. The system has two channels: Channel 1 is formed between the electrode in Region 1 (ABVN) and the lower electrode in Region 3, and Channel 2 is formed between the electrode in Region 2 (ATN) and the upper electrode in Region 3. **(F)** Roo system on a neonate.

tAN was administered using a unique wearable earpiece (Roo™ Therapy System, Spark Biomedical, Dallas, TX, USA) that targets the auricular branch of the vagus nerve (ABVN) and auriculotemporal nerve (ATN). This device recently received breakthrough device designation by the FDA. The system includes a disposable, multi-channel earpiece electrode and an External Pulse Generator (EPG) that are connected using a cable (Figure 2). The earpiece is worn on and around the left ear. The earpiece electrodes are made of hydrogel that is biocompatible and can remain on the infant’s ear for several treatment sessions without the need for replacement. Due to variations in the scalp and/or skull, certain instances required medical tape to hold the earpiece to the head. The addition of medical tape was well-tolerated in all infants.

tAN stimulation was delivered at 0.1 mA below perception intensity at two different areas using two discrete frequencies. Area 1/Channel 1 innervates the cymba concha region of the ear and targets the ABVN whereas Area 2/Channel 2 innervates the temporomandibular joint, anterior to the tragus region and targets the ATN. Stimulation was pre-programmed to a pulse-width of 250 μs. Channel 1 was programmed to a frequency of 5 Hz and Channel 2 was programmed to 100 Hz. The current intensity duty cycle was set to run for 5 min with a 10 s off period. Perception intensity for each area is independent and was determined by starting at 0.1 mA and increased by 0.1 mA until the infant responded to stimulation, usually by eye blinking or head turn. After the infant responded to stimulation, the current intensity was turned down by 0.1 mA and was set and to run for 30 min. When the 30-min tAN stimulation period was complete, the system was turned off and unplugged. The ear electrode may have been left on the infant if still adhering well to the skin.

tAN was administered up to four times per day for up to 12 days total. If the infant was weaned off morphine within 10 days, a 2-day tAN weaning period was implemented. On Day 1 of this weaning period, tAN was administered twice and on Day 2 tAN was administered once. All infants were observed for 48 h after cessation of morphine treatment and 24 h after cessation of tAN adjuvant therapy.

### Safety Outcome Monitoring

Safety measures included heart rate monitoring, pain assessment, and adverse event monitoring. Bradycardia (HR <80 bpm) was monitored using bedside neonatal intensive care unit cardiac monitors. The Neonatal Infant Pain Scale (NIPS) is a validated pain scale utilized in the NICU (Lawrence et al., 1993; Witt et al., 2016) was used to monitor pain levels and was recorded before and after the 30-min tAN stimulation period. There are six components to the NIPS: facial expression, crying, breathing patterns, arm and leg movements, and state of arousal. The NIPS scale scoring ranges from 0 to 7, with scores greater than 3 indicating discomfort. A score of 3 is similar to the pain level associated with a heel stick procedure to obtain blood and the maximum score of 6 is similar to a circumcision procedure without analgesia (Butler-O’Hara et al., 1998; Anand, 2001). Neonatal nurses routinely record NIPS scores throughout the day and infants with NOWS frequently have elevated NIPS scores. Thus, tAN stimulation was only halted or adjusted to a lower intensity if NIPS scores were 3 greater than baseline. The skin on the exterior and just inside the left ear was examined daily before electrode placement for redness and any signs of irritation. Stimulation of the right ear occurred if redness, signs of irritation, or any other issues were observed at the site of the electrode.

### Statistical Methodology

As this was a first-in-human prospective, single-arm, open-label trial, no formal statistical hypotheses were tested nor was the sample size derived using statistical principles. The sample size was based on an estimate of the number of infants that could be enrolled within a reasonable timeframe. Continuous data were summarized using descriptive statistics including *N* (number in analysis set), *n* (number of non-missing observations), mean, standard deviation, median, interquartile range (IQR), and range unless otherwise noted. Categorical variables were summarized using frequency counts and percentages. Paired sample *t*-tests were used to assess changes in heart rate.

## Results

### Demographics

Eight of the nine infants considered for participation were consented and enrolled in the study (parental refusal consent *n* = 1). Relevant infant and maternal demographics and baseline clinical characteristics are provided in Tables s 1, 2. These infants had a median gestational age of 38.2 weeks at enrollment and were exposed *in utero* to methadone, tobacco (tAN1); heroin, buprenorphine, cocaine, and tobacco (tAN2); buprenorphine (tAN3); opioids, methamphetamines, and benzodiazepines (tAN4); heroin, cocaine, and methadone (tAN5); heroin, methadone, tobacco, and THC (tAN6); hydromorphone (tAN7); and heroin, methamphetamines, methadone, and tobacco (tAN8). Additionally, tAN4 was on lorazepam and tAN6 was on clonidine at enrollment. The mean (SD) control oral morphine dose was 0.076 (±0.041) mg/kg administered every 3 h. Only three out of the eight infants (37.5%) received maternal breastmilk during the study. Half of the mothers were on a maintenance dose of methadone (daily dose ranging from 40 to 75 mg), 25% were on a maintenance dose of buprenorphine, and the remaining 25% were taking short-acting opioids.

**Table 1:** Infant and mother demographics.

Infants (*N* = 8)	
Median gestational age (range) in weeks	38.2 (35.1–43.9)
Median birth weight (range) in grams	2,810 (2,190–3,960)
Male sex—no. (%)	5 (62.5%)
Breast-feeding subgroup—no. (%)	3 (37.5%)
Median highest oral morphine dose (range) in mg/kg	0.06 (0.05–0.17)
Region 1 ABVN: Mean mA (SD) current intensity	0.32 (0.04)
Region 2 ATN: Mean mA (SD) current intensity	0.38 (0.14)
**Mothers (*N* = 8)**	
Use of methadone
Maintenance therapy—no. (%)	4 (50%)
Average daily dose (range) in mg	58.3 (40–75)
Use of buprenorphine—no. (%)	2 (25%)
Use of short-acting opioid—no. (%)	2 (25%)
Use of tobacco—no. (%)
Any	4 (50%)
5 c*igarettes a day*	
Drugs identified on meconium or infant drug screen—no. (%)
Cocaine	1 (12.5%)
Amphetamine	1 (12.5%)
Morphine	1 (12.5%)

**Table 2:** Baseline infant medication use and concomitant medical problems.

Participant	Medications	Concomitant medical problem(s)
tAN 1	Vitamin D	Hypoglycemia, resolved
tAN 2	Vitamin D Dolutegravir (5 mg) Lamivudine (10 mg/ml) Nystatin Sulfamethoxazole-Trimethoprim Zidovudine (10 mg/ml)	Early Latent Syphilis Herpes simplex virus infection Hepatitis C Gonorrhea Chlamydia
tAN 3	Vitamin D	Hyperbilirubinemia Abnormal x-ray of humerus Hyperosmolality/Hypernatremia Respiratory distress syndrome requiring initial CPAP
tAN 4	Ativan (0.05 mg/kg)	Meconium aspiration below vocal cords resolved
tAN 5	Penicillin Vitamin D	Hepatitis B (maternal) Hepatitis C (maternal) Ankyloglossia Syphilis (maternal) Tethered labial frenulum
tAN 6	Clonidine (7 mcg- >10 mcg) Simethicone (40 mg/0.6ml oral drop)	Tobacco use (maternal)
tAN 7	Gentamicin Ampicillin Ativan given once	Respiratory distress syndrome, resolved
tAN 8	Vitamin D Acetylcysteine Acyclovir Ampicillin Gentamicin	Hyperbilirubinemia Failed newborn hearing screen^1^ Hepatitis C (maternal) Hepatitis B (maternal) Lactobezoar

*^1^Failed routine newborn hearing screen, 8 days before starting tAN treatment. The infant had a formal hearing screen as an outpatient 9 days after discharge and passed the hearing screen at that time*.

### tAN Treatment

tAN was administered for an average (SD) of 9.5 (±2.6) days (range: 5–12 days) and the average (SD) number of tAN sessions was 30 (±9.5) (range: 13–43) across all eight infants. On average, infants received 3.1 (±0.4) tAN treatments per day.

### Safety

tAN therapy did not result in any unanticipated adverse events (device and non-device related) in any subject. One infant experienced redness at the stimulation site which resolved after 12 h on two separate occasions. Redness occurred after removing the hydrogel with sterile-water-treated gauze in most infants, but there was no irritation evident before the next treatment 3 h later, or after completion of tAN therapy in any infant.

Heart rate was monitored before tAN treatment, during tAN treatment, and after tAN treatment for bradycardia. Mean (SD) heart rate was 161.9 (±24.2) bpm prior to tAN treatment, 157.3 (±23.0) bpm during tAN treatment, 160.6 (±14.6) immediately after tAN treatment and 162.4 (±17.0) 15 min following tAN treatment. There were no observed episodes of bradycardia during or after tAN treatment and the mean heart rate was not significantly changed at any time point from the pre-tAN baseline value (paired samples *t*-test; *p* > 0.05).

The median NIPS score was 0 (IQR: 0.0–3.0) before tAN therapy and 0 (IQR: 0.0–2.0) after tAN treatment, suggesting minimal discomfort during stimulation. There were 26 sessions (12.0%) across the 217 total tAN sessions with concurrent NIPS score collection during which NIPS scores increased by 3 or more points from the pre-stimulation value during the post-tAN assessment.

### Morphine Administration Outcomes

**Figure 3:**
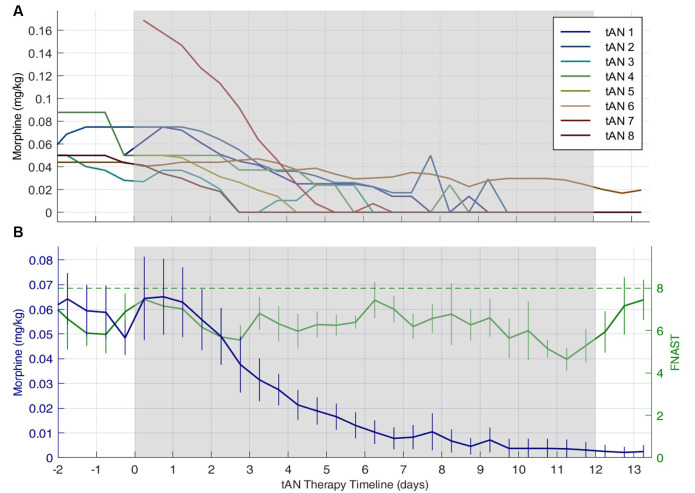
Individual and combined subjects daily finnegan neonatal abstinence scoring tool (FNAST) value and morphine dose plots. Daily morphine dose 2 days before, during, and 2 days post-tAN therapy. Gray shaded region indicates when tAN therapy was administered. Panel **(A)** represents individual subject morphine dose and **(B)** represents mean daily morphine dose across the entire cohort. In **(B)** the green dash line indicates the Finnegan score threshold requiring a change in morphine dose, the solid green line indicates mean FNAST scores, and the solid blue line indicates mean morphine dose. The number of days on oral morphine before the start of tAN therapy varied across all subjects but was accounted for in total morphine LOT. Data are means ± standard error of the means.

Figure 3 shows the mean daily morphine dose 2 days before, during, and 2 days post-tAN therapy for individual subjects (Figure 3A) and across the entire cohort (Figure 3B). The mean (SD) daily dose of morphine was 0.063 (±0.043) mg/kg at the start of tAN therapy, and morphine was consistently weaned every 12 h once tAN started. Table 3 provides both mean and median for total oral morphine LOT and oral morphine LOT after initiation of tAN treatment. The median oral morphine LOT was 9.0 days (IQR: 6.5–12.8; range: 4–43), and median LOT after tAN initiation was 6.0 days (IQR: 4.8–8; range: 3–16) (Table 3). One infant (tAN6) was enrolled after transfer from another facility at 3 weeks of age, after failing oral morphine replacement treatment. This infant skewed the mean (SD) total oral morphine LOT to 13.3 (±12.8) days and oral morphine LOT after initiation of tAN to 7.0 (±4.0) days. The number of days for both total oral morphine LOT and oral morphine LOT after tAN initiation for tAN6 were over two standard deviations above the mean, justifying exclusion from analyses. When tAN6 was excluded, median total oral morphine LOT and oral morphine LOT after tAN treatment stayed the same; however, mean (SD) total oral morphine LOT was similar to the median values at 9.0 (±4.7) days and oral morphine LOT after tAN at 5.7 (±1.9) days.

**Table 3:** Effectiveness outcomes (*N* = 8).

Outcome	Median (IQR)	Mean (SD)	Mean (SD) excludes tAN6^1^ (*N* = 7)	Range
Total oral morphine length of treatment (LOT) in days	9.0 (6.5–12.8)	13.3 (12.8)	9.0 (4.7)	4–43
Oral morphine length of treatment (LOT) after tAN initiation in days	6.0 (4.8–8)	7.0 (4.0)	5.7 (1.9)	3–16
Length of hospital stay in days	17.0 (15.3–28.5)	22.6 (12.5)	19.3 (8.9)	10–46

*^1^Number of days for both total oral morphine LOT and oral morphine LOT after tAN initiation were over 2 standard deviations above the mean for tAN6, justifying exclusion in analyses*.

During tAN therapy, all eight infants achieved average FNAST scores <8, indicating an overall reduction in withdrawal symptoms. All infants, except for one (tAN6), were completely weaned off morphine during tAN treatment. Following 12 days of tAN treatment, the mean daily dose of morphine for tAN6 was 0.018. The infant was completely weaned off morphine 4 days following tAN treatment. In tAN4, lorazepam weaning was initiated 1 day after discontinuing morphine. In total, five rescue morphine doses, resulting from FNAST scores >8, were administered across all subjects.

The median length of hospital stay, calculated as the number of days from date of birth to discharge (including all levels of care), across all eight infants was 17.0 days (IQR: 15.3–28.5; Range: 10–46) and the mean (SD) length of stay was 22.6 (±12.5) days.

## Discussion

In this prospective open-label trial, tAN was applied as an adjuvant therapy to oral morphine in neonates diagnosed with NOWS from *in utero* exposure to opioids including methadone, heroin, buprenorphine, and methamphetamines. tAN was administered 1 h before morphine administration, up to four times daily for up to 12 days. tAN as an adjuvant was safe, well-tolerated, and facilitated the successful rapid weaning of oral morphine in these newborns. The median LOT from the start of administering oral morphine (9 days) and the median LOT after tAN initiation (6.0 days) were both significantly lower than previously published data suggesting that the average NICU stay for infants undergoing pharmacotherapy for NOWS is 23 days (Patrick et al., 2015). Given that tAN treatment permitted rapid consistent weaning, our preliminary data suggests that using tAN as an adjuvant to pharmacotherapy could significantly reduce NICU stay. Furthermore, as indicated by the NIPS and adverse event profile, tAN did not produce any additional risks outside those observed with oral morphine. If these early results are confirmed, adjuvant tAN therapy compares favorably with the LOT and with the risk profile of oral opioid treatment as well as with other alternative non-invasive adjuvant NOWS treatments.

## Developing an Alternative to Opioid Replacement Therapy

Currently, no nationwide standard of care exists for managing NOWS (Patrick et al., 2014; Barlow et al., 2017). Treatment of NOWS usually follows a multimodal regime centered on controlled withdrawal and replacement drug therapy with oral morphine. However, newer opioid agents are being tested. The recent Blinded Buprenorphine OR Neonatal morphine solution (BBORN) trial reports the median length of treatment for oral morphine and sublingual buprenorphine as 28 and 15 days, respectively (Kraft et al., 2017). Furthermore, a randomized control trial that examined the safety and efficacy of methadone vs. morphine reported a median length of treatment of 15 and 11.5 days (Davis et al., 2018). However, treatments that reduce the need for neurotoxic opioids are a high priority in this vulnerable population (Flannery et al., 2020). The NEOPAIN trial, a large multicenter randomized study of 898 infants demonstrated that although morphine is effective in decreasing clinical signs of pain, it can cause significant acute adverse effects such as changes in the heart and respiratory rate, hypotension, nasogastric feeds, and need for intravenous supplemental nutrition (Anand et al., 2004; Attarian et al., 2014). Based on the vulnerability of the population and the potential for adverse effects, the authors of the NEOPAIN study suggested that morphine administration should be used judiciously and cautiously.

Furthermore, a review published in 2014 suggests that current protocols of morphine replacement in neonates alter critical development periods and can lead to adverse neuropsychological effects (Attarian et al., 2014). Specifically, morphine has been shown to affect apoptotic protein expression in animals and humans, suggesting that opioids alter various neurological pathways other than pain pathways. In support of the NEOPAIN study and the 2001 Consensus Statement for the Prevention and Management of Pain in the Newborn, the authors conclude that non-pharmacologic interventions should be a primary treatment option, and if unsuccessful, the use of opioid analgesics should be thoughtful and cautious. The use of opioid-based medication as the standard of care remains concerning due to the relatively high potential of compounding harm to the infants’ development and health.

Multiple studies have assessed the debilitating consequences of NOWS including otitis media and delayed cognitive and/or motor development (Rosen and Johnson, 1982; Kirkwood and Kirkwood, 1983). These consequences have been studied for decades. A study from 1982 comparing 38 neonates with NOWS to 23 healthy neonates found that neonates exposed to methadone prenatally had a significantly higher incidence of otitis media after an 18-month follow-up (Rosen and Johnson, 1982). A subsequent study in 1983 expanded on this finding demonstrating that recurrent otitis media is associated with persistent hearing loss and subsequent impairments in language skills (Kirkwood and Kirkwood, 1983). The authors concluded that children with recurring otitis media have a greater risk of learning disabilities which can have a substantial impact on the day-to-day function of both the child and caregiver. Additionally, NOWS has been shown to cause developmental cognitive delays based on the McCarthy Scales of Children’s Abilities, which assesses cognitive ability, including general cognitive index, perception, and memory. Children who had been exposed to heroin prenatally (*n* = 22) were shown to perform far worse on the McCarthy Scales when compared to their control counterparts (Wilson et al., 1979).

To assess motor development in infants, physicians use the Bayley Scale of Infant Development (BSID) to measure the current developmental functioning in areas of cognition, motor skills, and behavior. The BSID consists of two parts: the Psychomotor Developmental Index (PDI) and the Bayley Mental Developmental Index (MDI; Lennon et al., 2008). Multiple studies have shown that infants with NOWS have significantly lower PDI and MDI scores compared to healthy infants at 12, 18, and 24 months of age (Strauss et al., 1976; Rosen and Johnson, 1982; Johnson et al., 1984). A retrospective study also found that 3-year-old children (*n* = 28) with NOWS had significantly lower overall BSID composite scores compared to healthy children. A study focused specifically on the effects of buprenorphine found that 28 children (5–6 years of age) exposed to buprenorphine prenatally had significant problems with motor skills, memory, hyperactivity, impulsivity, and attention (Sundelin Wahlsten and Sarman, 2013).

Concerning non-pharmacologic treatment options, a protocol developed by the National Acupuncture Detoxification Association (NADA) has demonstrated effectiveness in reducing withdrawal symptoms in adults and has been recently utilized in newborns as an adjunctive treatment for NOWS (Raith et al., 2015; Weathers et al., 2015; Jackson et al., 2020). Raith et al. (2015) found that using the NADA protocol, handheld laser acupuncture is an effective adjunctive treatment for NOWS. More specifically, handheld laser acupuncture resulted in a significantly reduced duration of oral morphine and a significantly reduced length of hospital stay; the median length of treatment for morphine and morphine + laser was 39 and 28 days. Applying tAN using the Roo System in our cohort, also resulted in a decrease in the duration of oral morphine and overall days of treatment for each neonate. The similarity in these findings is likely due to the treatment location. The five acupoint regions targeted in the NADA protocol are innervated by two specific cranial nerve branches: the ABVN and the ATN (Round et al., 2013; Butt et al., 2020), the same cranial nerve branches targeted by the Roo System (Figures 2D–F), which uses hydrogel electrodes to deliver mild electrical stimulation.

## Proposed Mechanism of tAN

**Figure 4:**
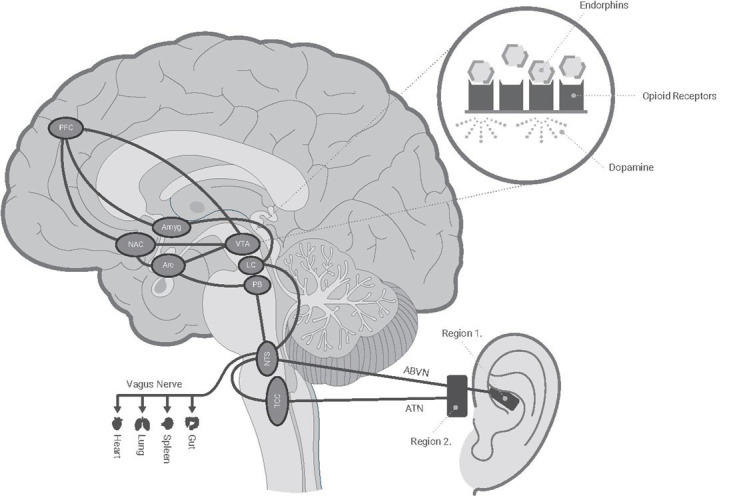
Schematic of hypothesized tAN innervation with central targets. Roo delivers individually tailored electrical stimulation targeting auricular cranial nerve branches of the ABVN (Vagus) and ATN (Trigeminal) nerves. These nerves send afferent signals to the NTS and TCC. The information is processed in the medulla and relayed to higher regions which may lead to the release of endogenous opioids (endorphins) and alleviation of opioid withdrawal symptoms. ABVN, auricular branch of the vagus nerve; Amyg, amygdala; Arc, arcuate nucleus; ATN, auriculotemporal nerve; LC, Locus coeruleus; NAC, nucleus accumbens; NTS, nucleus tractus solitarius; PB, parabrachial nucleus; PFC, prefrontal cortex; TCC, trigeminocervical complex; VTA, ventral tegmental.

Functional magnetic resonance imaging studies have examined the effects of ABVN stimulation on brain network activity. In comparison to earlobe stimulation, ABVN stimulation activates the nucleus tractus solitarius, locus coeruleus, spinal trigeminal nucleus, parabrachial area, periaqueductal gray, amygdala, and nucleus accumbens (Frangos et al., 2015; Yakunina et al., 2017; Badran et al., 2018b; Kaniusas et al., 2019; Qureshi et al., 2020). An earlier study delivered direct electrical stimulation to the parabrachial area (PBA) and arcuate horn (ARH) regions which triggered the release of endogenous opioids (endorphins), allowing for analgesic effects (Han et al., 1991). Importantly, this analgesic effect was demonstrated to be dependent on stimulation frequency. Whereas the ARH provided optimal analgesic effect at lower frequencies (16–32 Hz), and PBA at higher frequencies (64–128 Hz), respectively. Figure 4 illustrates a possible mechanism for tAN as a method to activate the endorphinergic system *via* modulation specific brain regions. As an example, endorphins can bind to the opioid receptors in the ventral tegmental area activating dopaminergic neurons, and potentially leading to amelioration of pain and withdrawal symptoms (Han et al., 1991; Han and Wang, 1992; Oleson, 2002; Sator-Katzenschlager and Michalek-Sauberer, 2007; Meade et al., 2010). Additionally, the locus coeruleus (LC)-norepinephrine system plays a role in dependence/addiction and is a primary target for multiple substances of abuse, including opioids (Valentino and Volkow, 2020). During a state of stress, LC neurons are hyperactive, which leads to hallmark symptoms of withdrawal: hyperarousal and insomnia (Aghajanian, 1978; Ivanov and Aston-Jones, 2001). Endorphins that bind LC receptors can attenuate LC excitation, and ultimately alleviate withdrawal symptoms. Furthermore, α2-adrenergic antagonists (clonidine and lofexidine) suppress LC activation and are frequently used clinically to reduce opioid withdrawal. Thus, the physiological effects we observe support our hypothesis that tAN may be modulating these specific systems. However, further studies are needed to elucidate the therapeutic mechanism.

## Limitations and Future Directions

This first-in-human study was open-label with no sham control group, limiting interpretation of the study results in terms of the placebo effect (placebo effect by proxy) and true treatment effect. Although there is still debate as to whether the placebo effect exists in infants and to what degree (Barbier et al., 1994; Paul et al., 2014; Colloca, 2015; Kossowsky and Kaptchuk, 2015), a randomized, sham-controlled study is warranted to further explore treatment effect of adjuvant use of tAN during oral morphine weaning. Although the therapeutic benefits appear to be clinically meaningful, an RCT design would further address additional study limitations, namely the small sample size and conduct at a single center.

The inclusion of infants at different durations of morphine therapy does not allow for interpretation of the potential effect of tAN during early administration of morphine therapy (e.g., different levels of opioid withdrawal). However, the results of this study may be more representative of real-world clinical utilization of tAN therapy given the inclusion of infants at different durations of morphine therapy.

Lastly, our approach to tAN therapy included stimulation of the left AVBN and ATN. Both vagal and trigeminal branches have been targeted for pain therapy (Miller et al., 2016). Interestingly, both vagal and trigeminal afferents synapse on to the periaqueductal gray (Benarroch, 2012), stimulation of which, in humans, has shown to release endogenous opioids (Sims-Williams et al., 2017). Given these findings, we hypothesized that stimulation of vagal as well as trigeminal activation would synergistically mediate endogenous opioid release. However, this clinical trial was not designed to test these two working hypotheses. Further investigation into physiological biomarkers of opioid withdrawal and the mobilization of endogenous opioids may optimize tAN therapy. Although *in vivo* testing in human neonates is limited by ethical constraints (i.e., positron emission tomography scanning with radiotracers for opioid receptors), we may test salivary cortisol as a non-invasive measure of stress and measure oxidative stress in the brain *via* non-invasive magnetic resonance spectroscopy (Moss et al., 2018). While both of these markers are associated with opioid withdrawal (Ward et al., 2020), they are not direct measures of endogenous opioid release by tAN vs. another mechanism. Therefore, precise elucidation of mechanism relies on laboratory models of NOWS or pain models of neuromodulation of these cranial nerves.

## Conclusion

This is the first study investigating the effects of tAN, as adjunctive therapy to oral morphine, in the reduction of opioid withdrawal signs and symptoms in newborns with NOWS. Across all study participants, tAN was shown to be safe, well-tolerated, and seemed to facilitate the rapid weaning of oral morphine. The results also suggest that tAN may provide alleviation of withdrawal symptoms associated with NOWS. If proven safe and effective in future trials, tAN may expand non-pharmacological treatment options for these infants.

## Data Availability Statement

The raw data supporting the conclusions of this article will be made available by the authors, without undue reservation.

## Ethics Statement

The studies involving human participants were reviewed and approved by Medical University of South Carolina (MUSC) Institutional Review Board. Written informed consent to participate in this study was provided by the participants’ legal guardian/next of kin.

## Author Contributions

BB, DJ, GO’L, AC, NK, and SW made substantial contributions to conception and design, acquisition of data, or analysis and interpretation of data, participated in drafting, editing, and final approval of this manuscript. All authors contributed to the article and approved the submitted version.

## Conflict of Interest

BB and DJ are named inventors on brain stimulation patents/devices assigned to the Medical University of South Carolina. BB has equity in Bodhi NeuroTech, Inc. NK, SW, and AC are employees and shareholders of Spark Biomedical, Inc. The remaining author declares that the research was conducted in the absence of any commercial or financial relationships that could be construed as a potential conflict of interest.
